# Supplementary material for: Comparative 3D ultrastructure of Plasmodium falciparum gametocytes
Source: Nat Commun. 2025 Jan 2;16:69. doi: 10.1038/s41467-024-55413-5 (PMC11695595; doi:10.1038/s41467-024-55413-5)
Supplement: Supplementary file 1 — Supplementary Info [file 41467_2024_55413_MOESM1_ESM.pdf]

Supplementary Figures

Comparative 3D ultrastructure of *Plasmodium falciparum* gametocytes

Felix Evers[1], Rona Roverts[2,3], Cas Boshoven[1], Mariska Kea-te Lindert[2,3], Julie M.J. Verhoef[1], Nico Sommerdijk[2,3], Robert E. Sinden[4], Anat Akiva[2,3], Taco W.A. Kooij[1*]

[1]Department of Medical Microbiology, Radboudumc Center for Infectious Diseases, Radboud University Medical Center, Nijmegen, The Netherlands.
[2]Electron Microscopy Center, RTC Microscopy, Radboud University Medical Center, Nijmegen, the Netherlands.
[3]Department of Medical Biosciences, Radboud University Medical Center, Nijmegen, the Netherlands.
[4]Department of Life Sciences, Imperial College London, London, UK.

* taco.kooij@radboudumc.nl

Note S1 Membrane contact sites

Membrane contact sites (MCS) have typically been defined as areas of membranes of two organelles that are in close proximity to each other. These areas can be homotypic (between the same type of organelle, e.g. mitochondrion-mitochondrion MCS) or heterotypic (between different organelle types, e.g. mitochondrion-ER MCS). To increase clarity and create a unified vocabulary Scorrano *et al.*[112] have put together a set of unifying features that a MCS should have to be characterized as such.

1. There should be tethering forces from either protein-lipid or protein-protein interactions that maintain the distance. This replaces previous proximity ranges that were used as guidelines as contact sites have been found that far exceed these distance guidelines.

2. There should be no fusion of membranes. If there is fusion of membranes this can be referred to as docking or fusion. Vesicular transport at the contact site may exist within this definition.

3. All contact sites should fulfill a specific function. A disruption of a contact site should impact cell function.

4. MCS should have a defined proteome or lipidome that is required for their function, maintenance, or regulation.

5. Time is not a factor. Dynamic or transient structures that fulfill the other requirements can be considered a MCS.

A

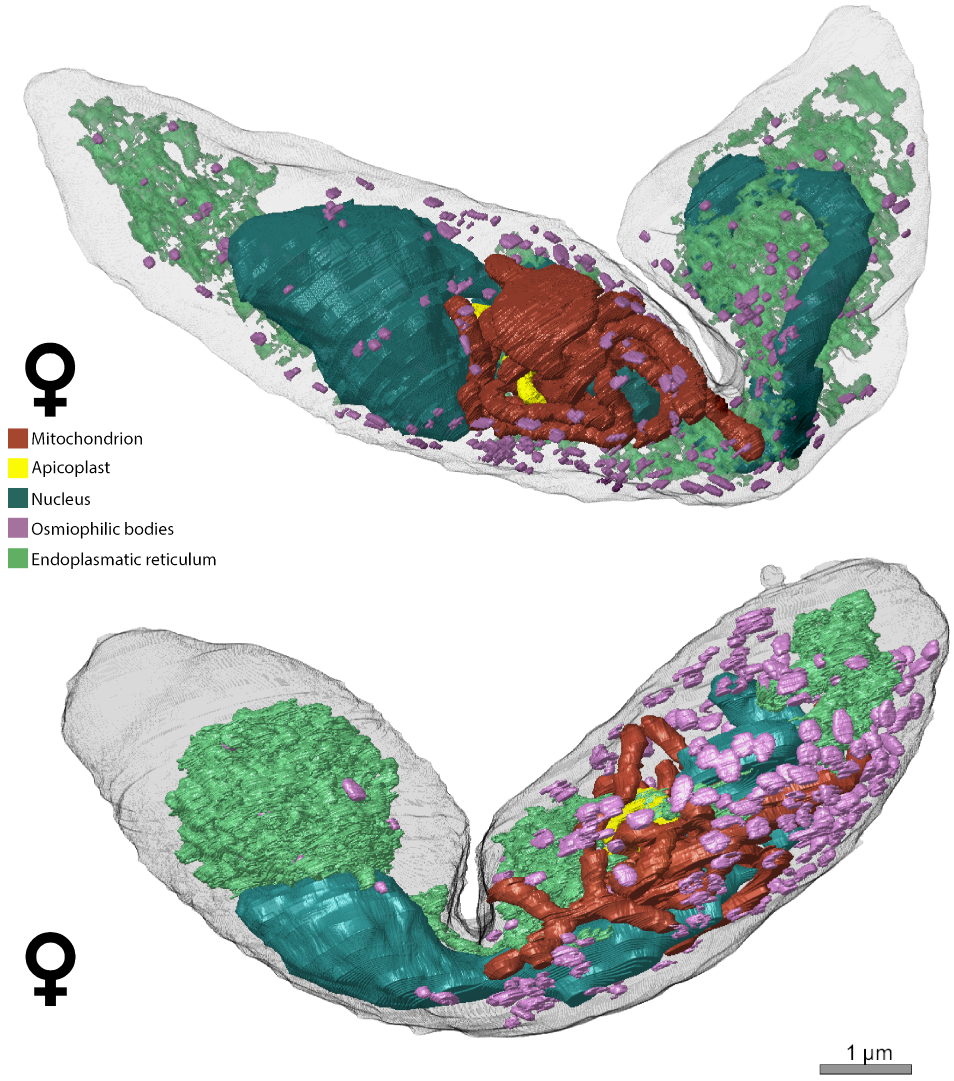

B

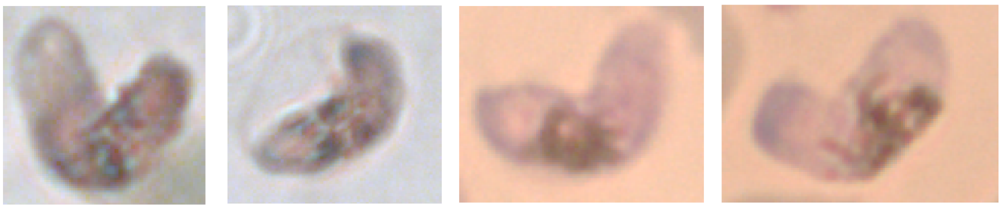

Figure S1. **Ultrastructural features of contorted mature female gametocytes.**
A. Renderings of two contorted mature female gametocytes with a typical contorted appearance observed in gametocyte cultures. Ultrastructural features shown include nucleus, mitochondrion, ER, Golgi, osmiophilic bodies, and apicoplast. **B.** Exemplar crops from Giemsa-stained smears of gametocyte cultures showing mature female gametocytes with similar knicked appearances.

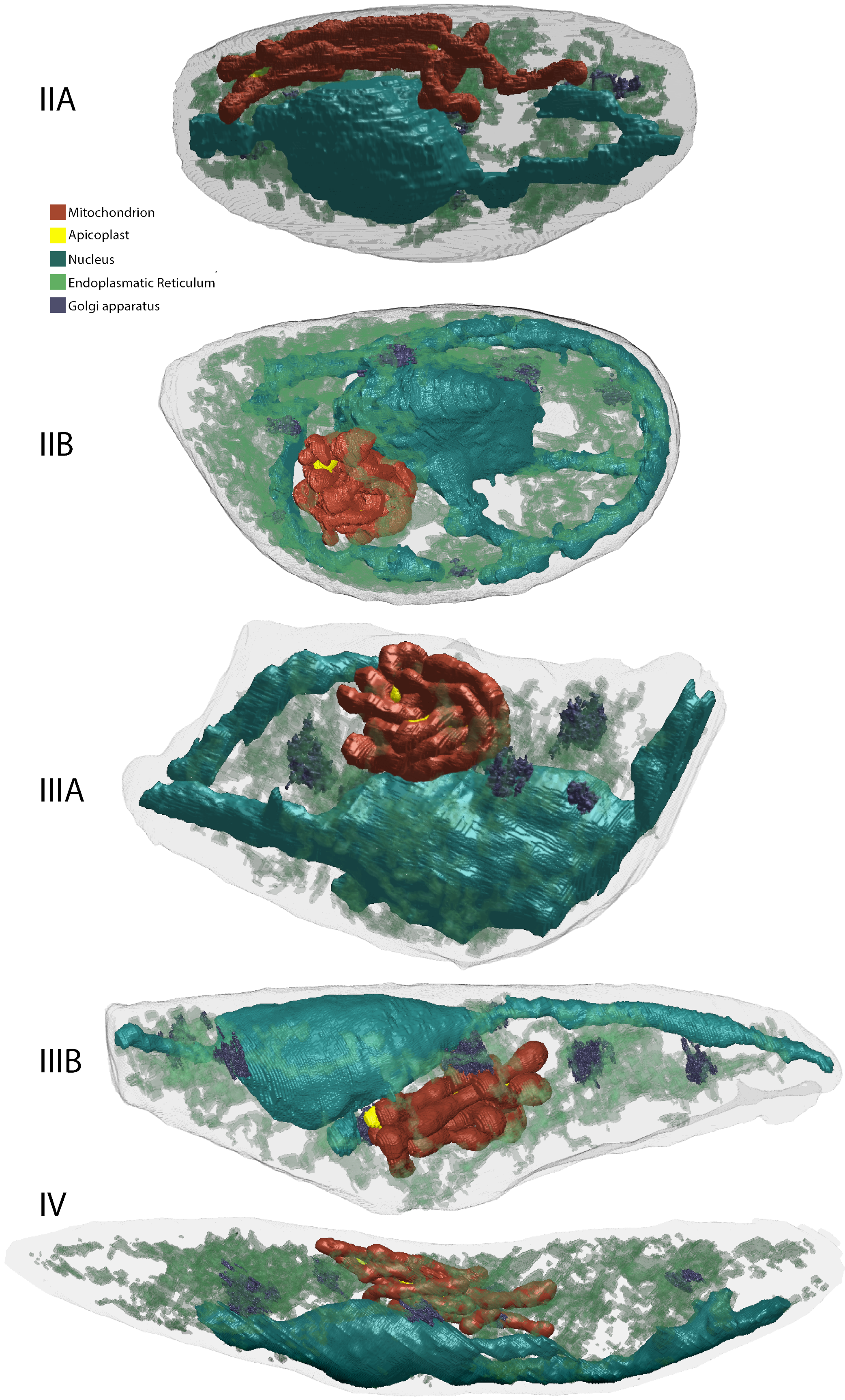

Figure S2. **Ultrastructural features of gametocytes in different developmental stages.**
Renderings of gametocytes in different developmental stages showing nucleus, mitochondrion, ER, Golgi apparatus, and apicoplast. ER rendering was made slightly transparent to allow better visibility of other features. Classifications were made based on cellular features typically associated with the respective developmental stages such as IMC cover or morphology of the parasite. Exemplary parasites were used for rendering but features were consistent across all datasets.

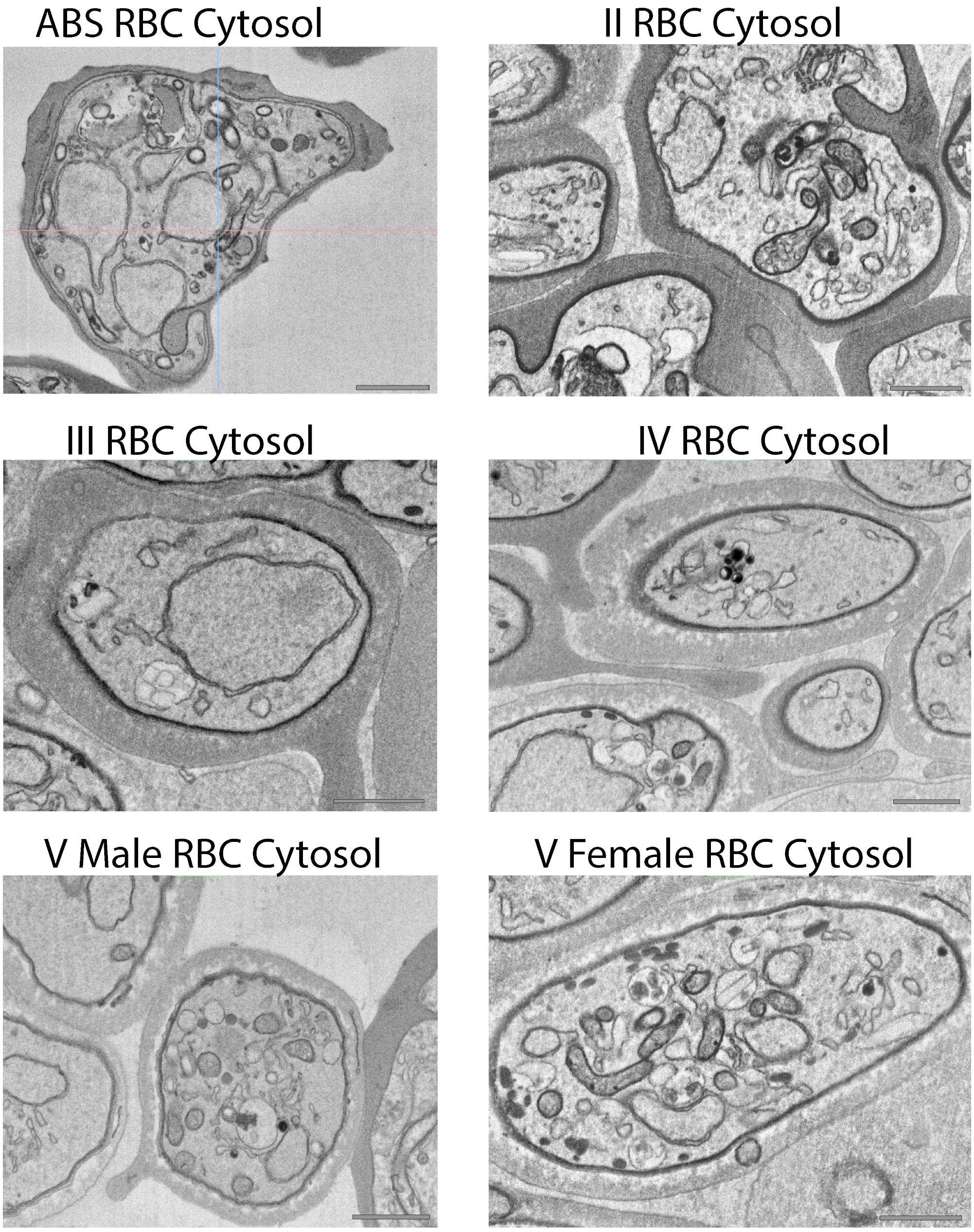

Figure S3. **Differing appearance of red blood cell cytoplasm in asexual blood stages and different gametocyte developmental stages.**

RBCs infected with ABS and stage II gametocytes contain a homogenous and relatively more electron dense cytoplasm**.** Starting in stage III the cytoplasm becomes increasingly electron lucent and an even more electron lucent corona starts to develop around and in close proximity to the gametocyte. Exemplary parasites were used for rendering but features were consistent across all datasets.

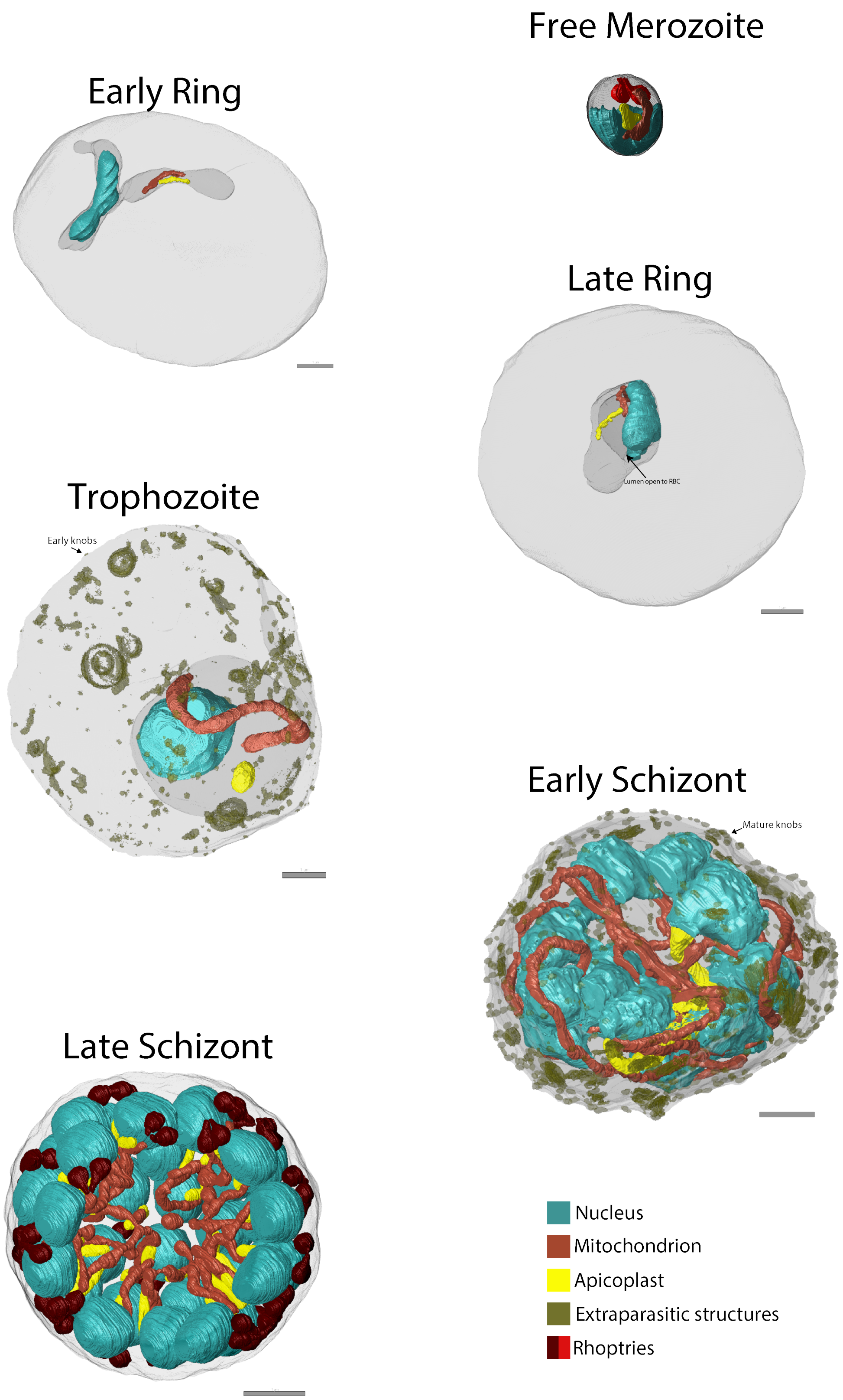

Figure S4. **Ultrastructural features of asexual blood stages**

Renderings of an extracellular merozoite, an early and late ring-stage parasite, a trophozoite, and an early and late schizont. Nuclei, mitochondria, and apicoplasts are shown for all parasites. For all cells except the free merozoite and late schizont, the outline of the infected RBC is shown. Extraparasitic structures in the RBC cytosol are only shown in the trophozoite and early schizont as they are absent in the ring stages while the RBC is not shown for the late schizont. Rhoptries are only

present in the late schizont and merozoite. Exemplary parasites were used for rendering, but features were consistent across all datasets.

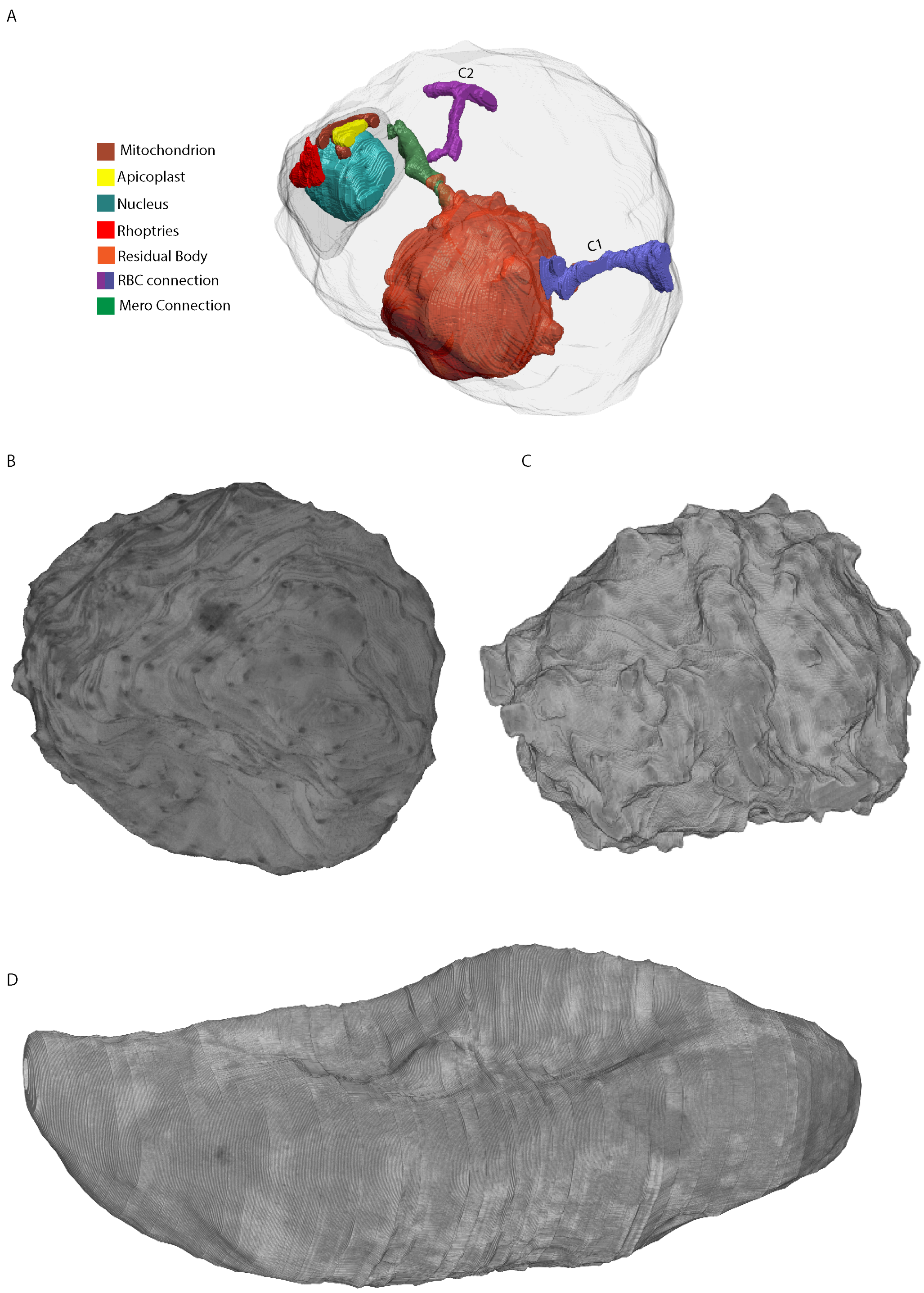

Figure S5. **Additional features of asexual blood stages.**
A. A segmented schizont harbors long tubular connections between the residual body and the RBC cytoplasm (C1, C2) and a daughter merozoite. **B-D.** Morphology of RBC infected by a developing schizont, a segmented schizont, and a mature gametocyte with gray values from the underlying EM data. Exemplary parasites were used for rendering but features were consistent across all datasets.

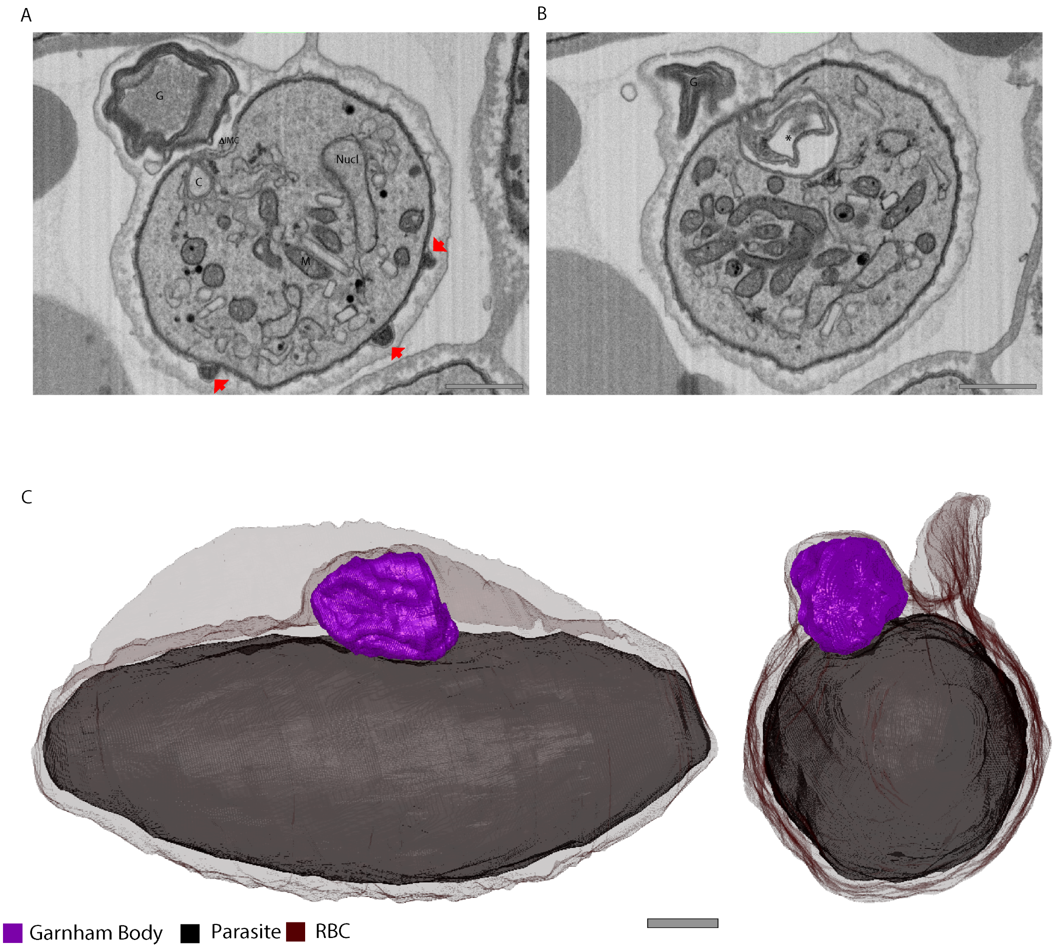

Figure S6. **The Garnham body.**
A. Exemplar micrograph showing membrane makeup of the Garnham body (G) and the presence of a cytostome (C), local lack of IMC (ΔIMC) and cellular protrusion (arrow). **B.** Exemplar micrograph showing aberrant internal structure opposing the Garnham body in the RBC (*). **C.** Rendering of the Garnham body from two angles. Scale bars = 1 μm.

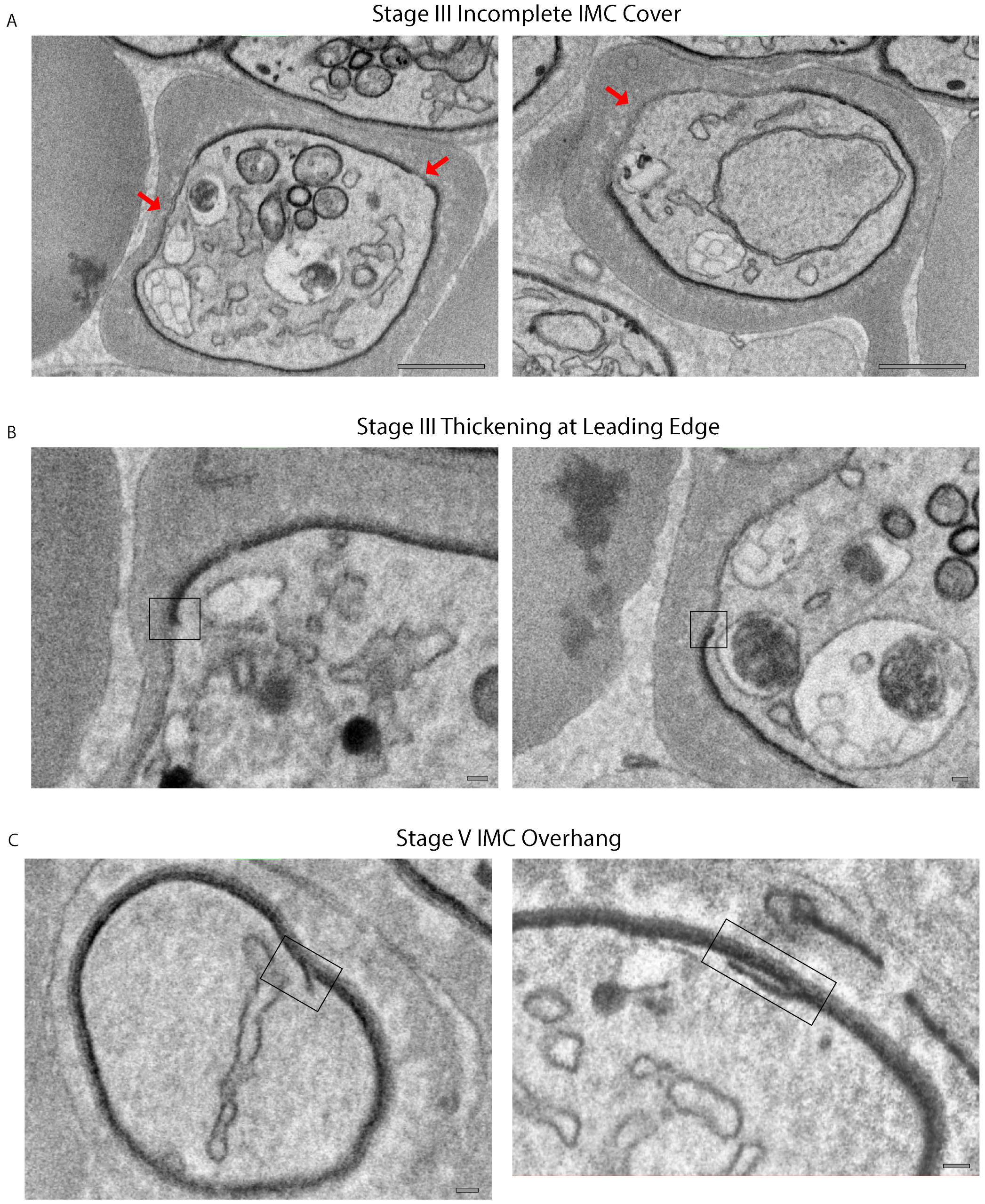

Figure S7. **Inner membrane complex features in stage III and stage V gametocytes.**
A. Micrographs with examples of incomplete IMC cover in stage III gametocytes highlighted with arrows. Scale bars = 1 µm. **B.** Putative local thickening of IMC as described by Schneider *et al.* within boxes. Scale bars = 0.1 µm. **C.** IMC overhangs observed at polar ends of mature gametocytes. Scale bars = 0.1 µm. Exemplary parasites were used but features were consistent across all datasets.

Canonical Cytostome in ABS

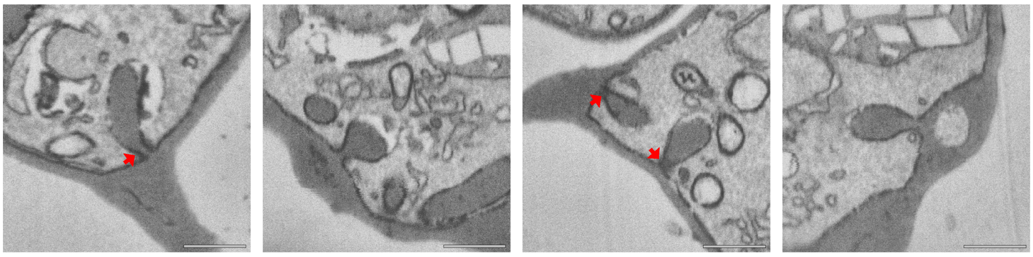

Figure S8. **The canonical cytostome.**
Exemplar micrographs of the cytostome of trophozoites and schizonts. Arrows highlight the cytostomal collar. Scale bars = 1 µm.

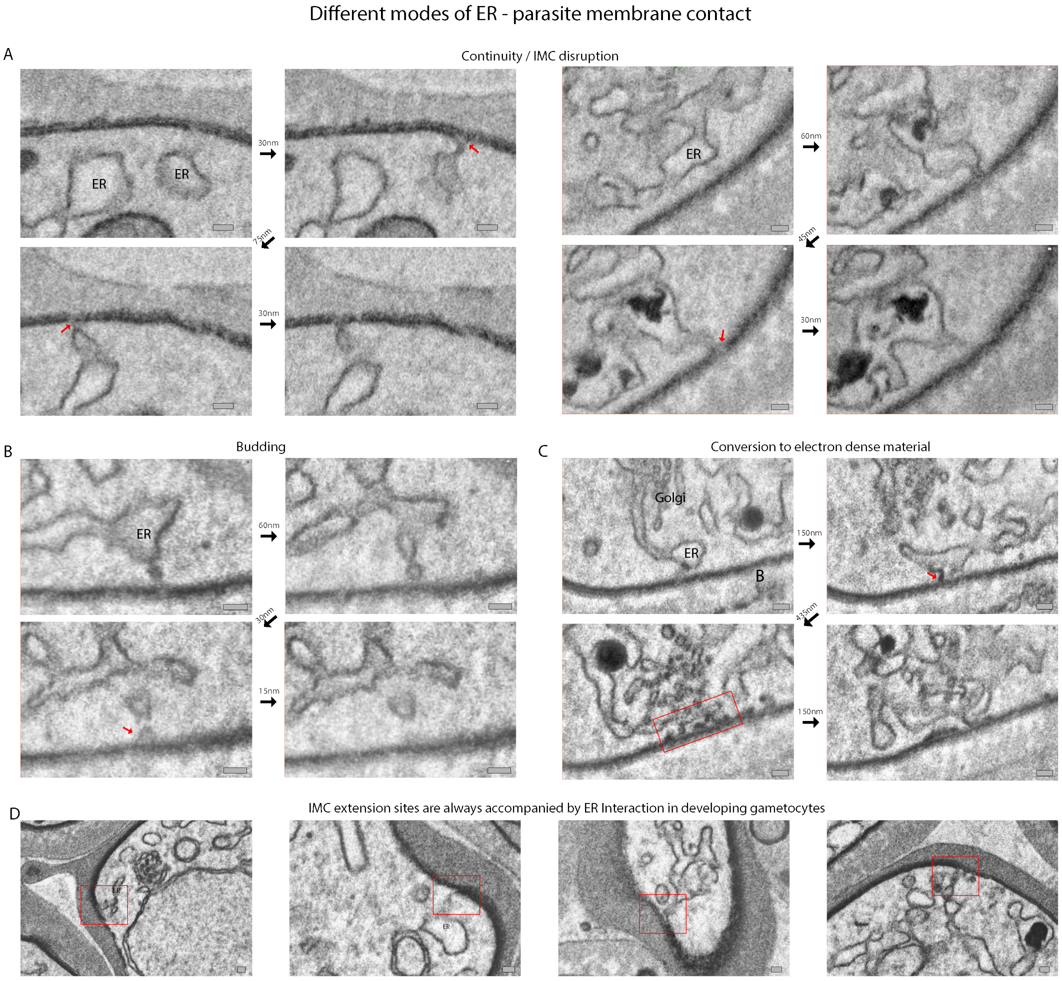

Figure S9. **Interactions between endoplasmic reticulum and parasite membrane.**

A-C. Series of micrographs from mature gametocytes showing ER in different putative interactions with the parasite membrane. **A.** The ER directly contacts and is continuous with the IMC, leading to a local disruption of the IMC. **B.** Same as (A) but connecting piece seemingly buds off from ER. **C.** ER is continuous with electron dense material that contacts the IMC. Black arrows between images express distance in the z-axis (acquisition plane) from one slice to the other. **D.** Exemplar micrographs showing contact of ER with four IMC extension sites that could be identified in a single developing gametocyte. All scale bars = 0.1 µm.

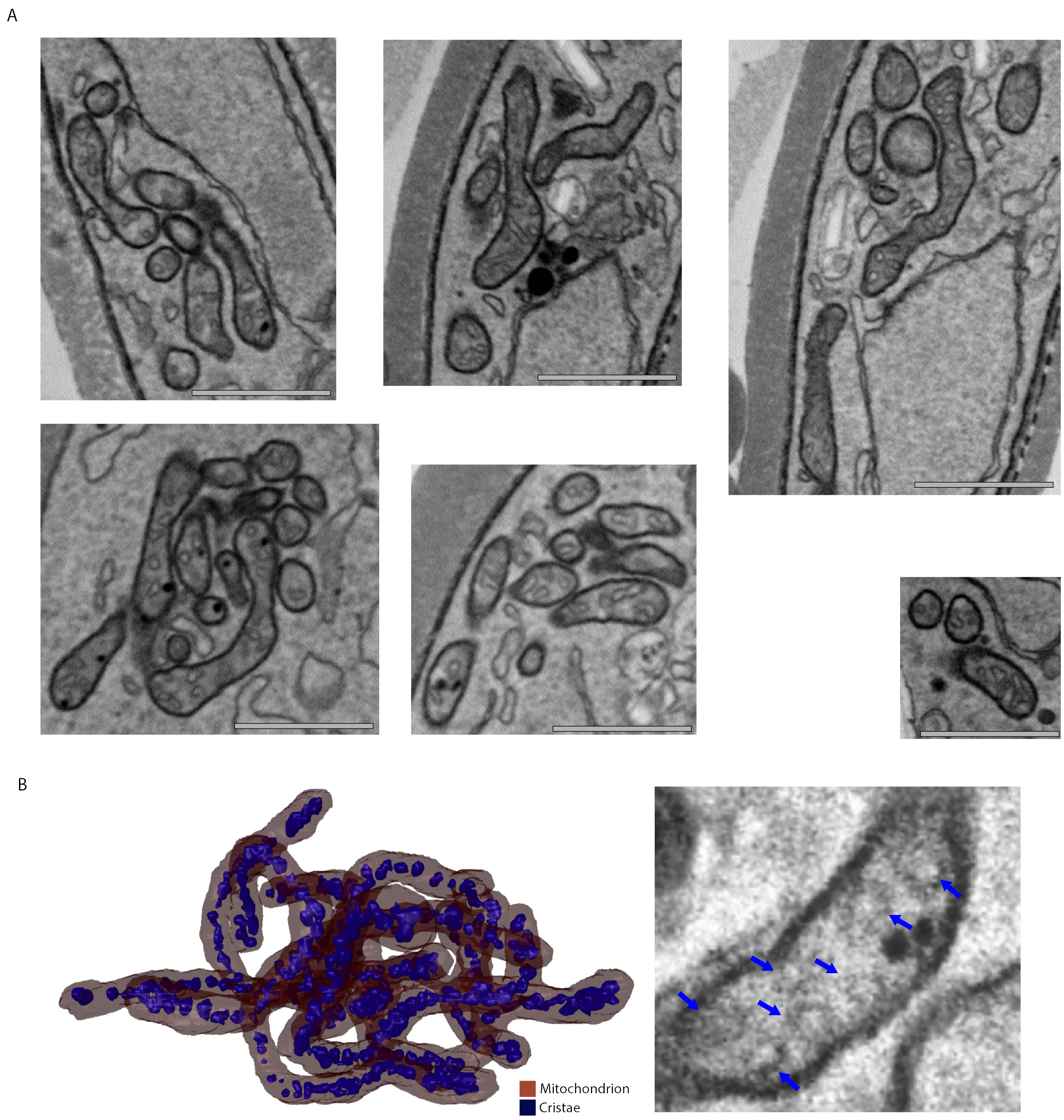

Figure S10. **Appearance and distribution of cristae in gametocytes.**
A. Exemplar micrographs from the low-noise gametocyte serial sectioning data. Note the presence of apparent branching points and proximity between individual cristae. The apicoplast often contained within a mitochondrial cluster is clearly distinct due the lack of internal membranous structures. Scale bars = 1 µm. **B.** Rendering of cristae (blue) within a gametocyte mitochondrion (red). Appearance of cristae in FIB-SEM data is highlighted in an exemplar micrograph through arrows.

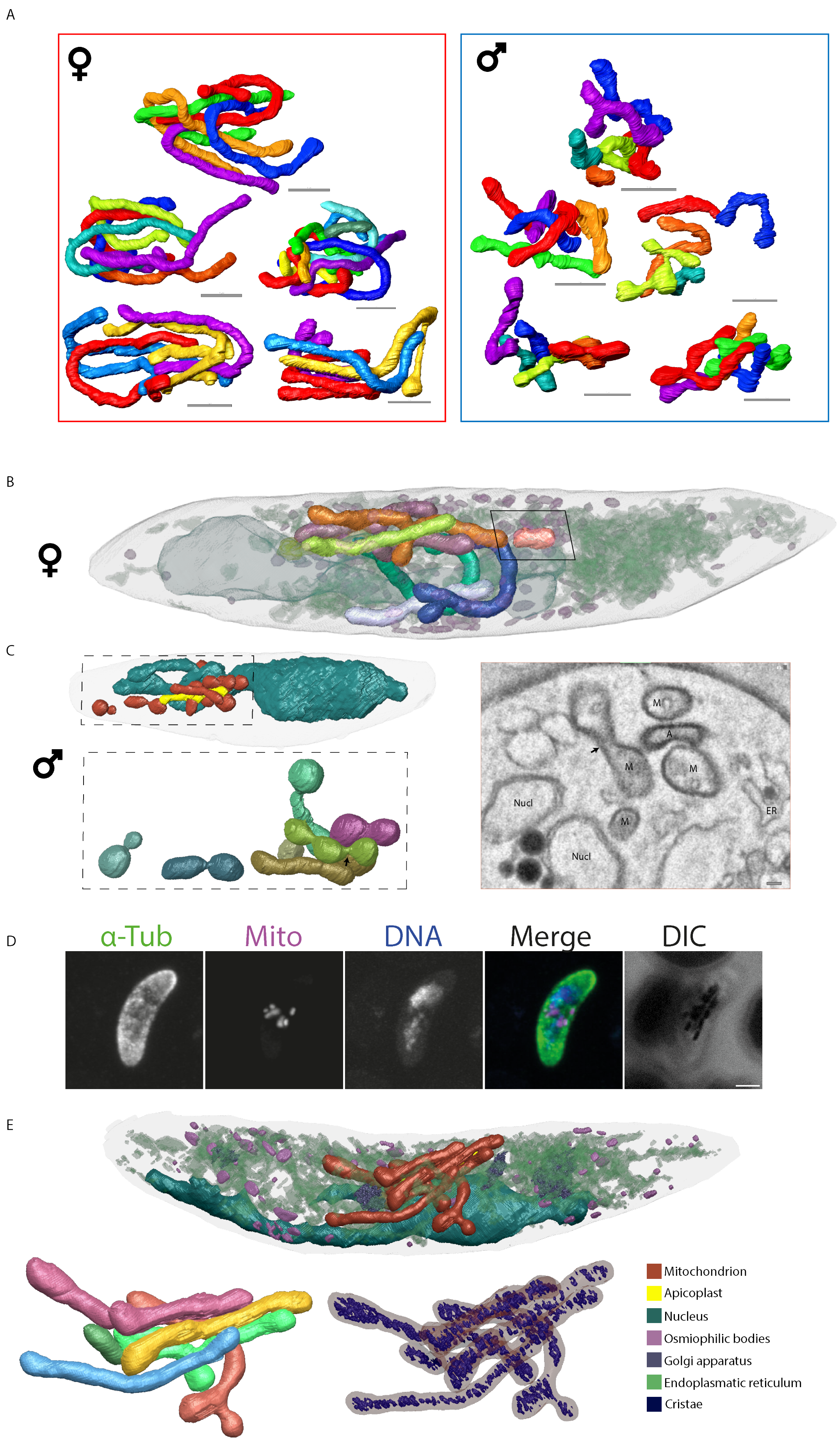

A
♀
♂
B
♀
C
♂
M
A
M
M
Nucl
M
ER
Nucl
D
α-Tub
Mito
DNA
Merge
DIC
E
Mitochondrion
Apicoplast
Nucleus
Osmiophilic bodies
Golgi apparatus
Endoplasmatic reticulum
Cristae

Figure S11. **Elaboration on multiple mitochondria in gametocytes.**

A. Renderings of mitochondrial clusters from female (red box) and male (blue box) mature gametocytes. Each color depicts a separate mitochondrion Scale bar = 0.5 µm **B.** Rendering of a stage IV female gametocyte. Each of the seven mitochondria rendered in a separate color. One small mitochondrion is clearly recognizable as removed from the remainder (black rectangle). Nucleus, osmiophilic bodies, ER, and apicoplast are rendered with high transparency to provide cellular context. **C.** Rendering of nucleus, apicoplast, and dispersed mitochondria (top left), a zoom of only mitochondria without cellular context (bottom left), and an exemplar micrograph showing atypical mitochondrial morphology and constriction site (black arrow). **D.** Immunofluorescence analysis of a mature male gametocyte. Depicted left to right are maximum intensity projections using anti-α-tubulin antibodies, MitoTracker™ , DAPI, merge of all channels and differential interference contrast (DIC) images. α-Tubulin was used to distinguish male and female gametocytes, DNA was visualized using DAPI, and mitochondria were visualized using MitoTracker. Mitochondrial staining suggests presence of multiple dispersed mitochondria. Scale bar = 2 µm. **E.** Renderings of a stage IV female gametocyte (upper section) and its mitochondrion highlighting the multiple mitochondria through different colors and distribution of cristae in a transparent mitochondrion (lower section).

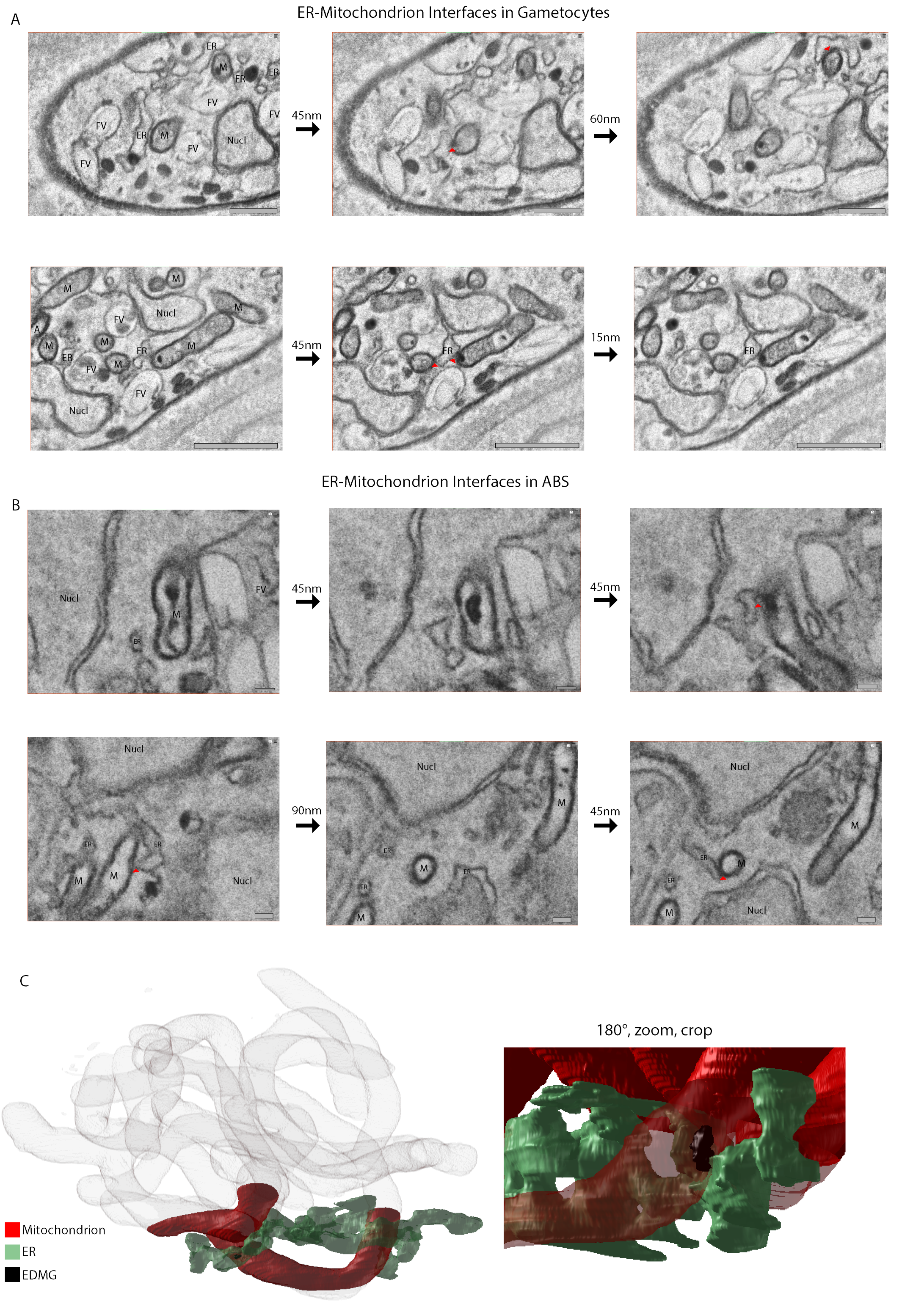

Figure S12. **Endoplasmic reticulum-mitochondrion interfaces in gametocytes.**
Series of micrographs showing EDMGs spanning across mitochondrion and ER in **A**. gametocytes, scale bars upper panel = 0.5 µm, scale bars lower panel = 1 µm, and **B**. ABS, scale bars = 0.1 µm. **C.** Rendering of EDMG-ER-mitochondrion connection.

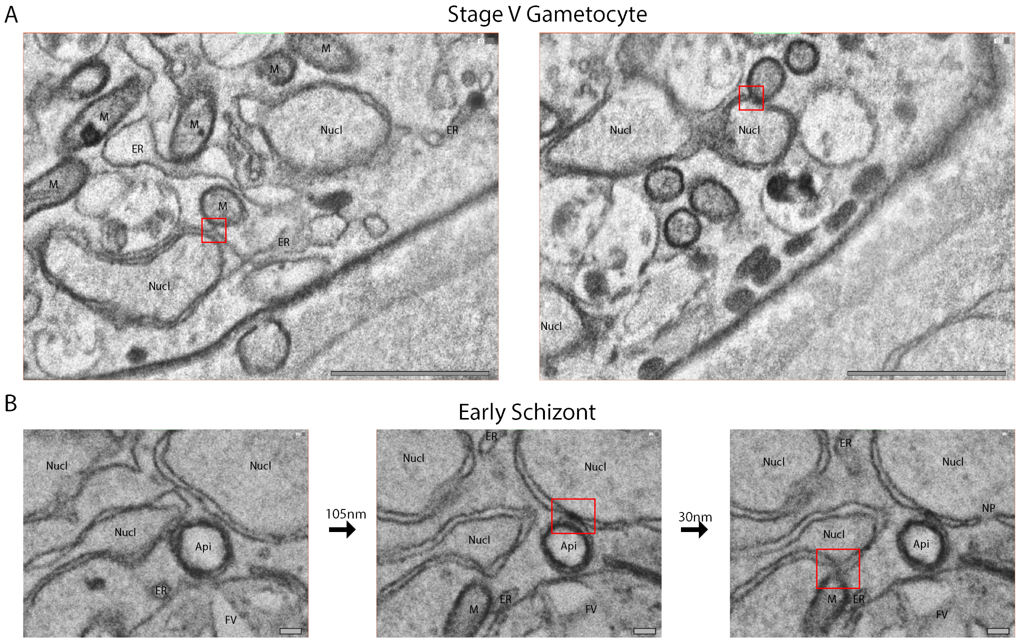

Figure S13 | **Putative organelle interfaces in different blood stages.**
A. Representative micrographs showing putative mitochondrion-nucleus interaction sites in a mature gametocyte. The respective sites are highlighted through a red box. Scale bars = 1 µm. **B.** Representative series of micrographs showing putative mitochondrion-nucleus and apicoplast-nucleus interaction sites in a schizont. The three frames are crops at the same x,y-coordinates in the acquisition plane but are at different z-depth with the respective distance displayed between micrographs. Scale bars = 0.1 µm M = Mitochondrion; ER = Endoplasmic reticulum; Nucl = Nucleus; FV = Food vacuole.

A

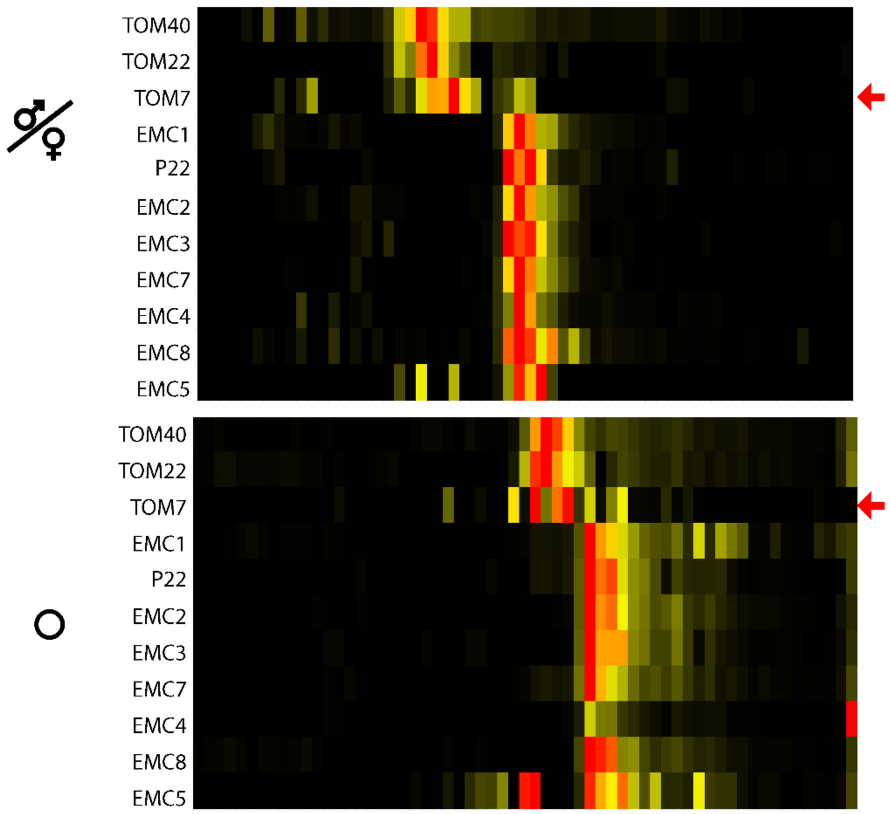

B

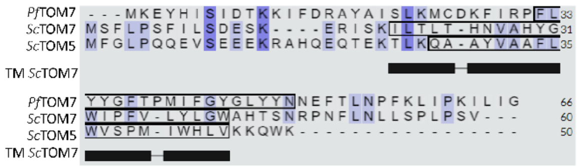

C

	*Pf*TOM7	*Sc*TOM7	*Sc*TOM5
*Pf*TOM7	100.00%	15.79%	12.77%
*Sc*TOM7	15.79%	100.00%	26.09%
*Sc*TOM5	12.77%	26.09%	100.00%

D

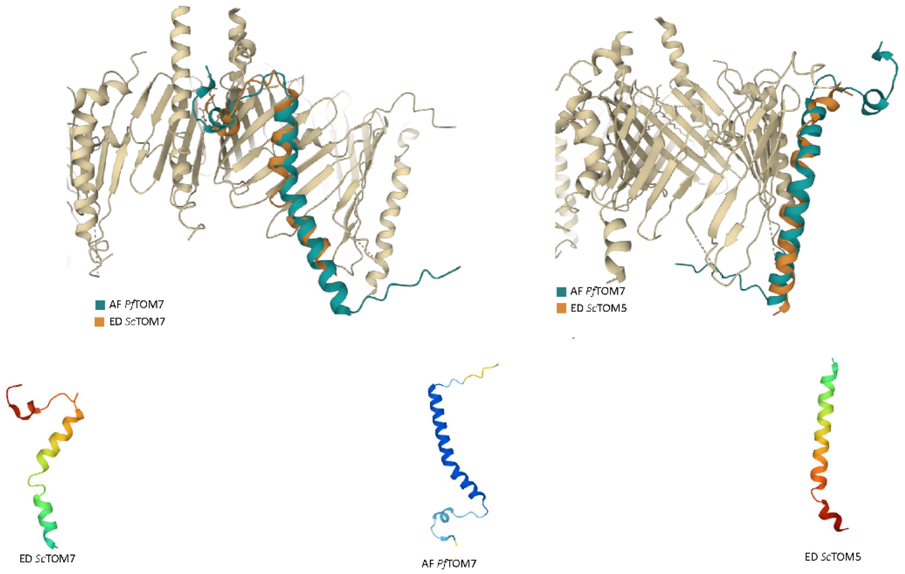

Figure S14. ***Pf*TOM7 as a putative endoplasmic reticulum membrane complex interactor and membrane contact site facilitator.**

A. Heatmap showing migration of proteins (rows) in native gel electrophoresis (columns). High relative protein abundance is indicated. Appearance in the same column suggests overlapping migrations patterns and putative assignment to a protein interaction. Upper heatmap is derived from a gametocyte sample, lower heatmap is derived from an ABS sample. **B.** Multiple sequence alignment of *Pf*TOM7 with *Sc*TOM7 and *Sc*TOM5. Conserved residues have a purple background and residues with light-blue background are conserved in 2/3 sequences. Black boxes show presence of TM domains known from *Sc*TOM7. **C.** Sequence similarity matrix showing sequence identity between *Pf*TOM7, *Sc*TOM7, and *Sc*TOM5. **D.** Structural alignment of the *Pf*TOM7 AlphaFold prediction with the experimentally determined structures of *Sc*TOM7 and *Sc*TOM5 in the context of the experimentally determined structure of the TOM complex.
