# Supplementary material for: Comparative 3D ultrastructure of Plasmodium falciparum gametocytes
Source: Nat Commun. 2025 Jan 2;16:69. doi: 10.1038/s41467-024-55413-5 (PMC11695595; doi:10.1038/s41467-024-55413-5)
Supplement: Supplementary file 2 — Description Of Additional Supplementary File [file 41467_2024_55413_MOESM2_ESM.pdf]

Description of additional supplementary files

Supplementary movie 1

Description: Animation of female GCT rendered in Fig. 1A. The various organelles are highlighted and shown from various angles and with the same color-coding as in Fig. 1A. The red blood cell surrounding the gametocyte is also shown in light red.

Supplementary movie 2

Description: Animation of female GCT rendered in Fig. 1B. The various organelles are highlighted and shown from various angles and with the same color-coding as in Fig. 1B. The red blood cell surrounding the gametocyte is also shown in light red.

Supplementary movie 3

Description: Animation of early schizont rendered in Fig. 1C. The various organelles are highlighted and shown from various angles and with the same color-coding as in Fig. 1C.

Supplementary movie 4

Description: Aligned image stack of electron micrographs of a mature gametocyte. Overlayed on the micrographs are mitochondria outlines (orange) and outlines of the cristae (blue). Mitochondria appear cristate across their whole volume and segmentations suggest interconnectivity between cristae. Scale bar = 1 µm

Supplementary movie 5

Description: Animation of female gametocyte mitochondria shown in Fig. 6A. First distribution of cristae across the mitochondrion is shown in 3D and rotated and then the individual mitochondria of the mitochondrial cluster are shown sequentially to make their lack of direct connectivity clear.

Supplementary movie 6

Description: Animation of male gametocyte mitochondria shown in Fig. 6A. The individual mitochondria of the mitochondrial cluster are shown sequentially to make their lack of direct connectivity clear.

Supplementary movie 7

Description: Animation of IFA data from a female gametocyte Fig. 6D column 1. First intensity data is shown with same color matching as Fig 6D. Then segmentation mask is overlayed on the mitochondrial marker channel and rotated showing the apparent presence of multiple mitochondria.

Supplementary movie 8

Description: Animation of IFA data from a female gametocyte Fig. 6D column 2. First intensity data is shown with same color matching as Fig 6D. Then segmentation mask is overlayed on the mitochondrial marker channel and rotated showing the apparent presence of a single mitochondrion.

Supplementary movie 9

Description: Animation of IFA data from a male gametocyte Fig. 6D column 3. First intensity data is shown with same color matching as Fig 6D. Then segmentation mask is overlayed on the mitochondrial marker channel and rotated showing the apparent presence of multiple mitochondria.

Supplementary movie 10

Description: Animation of IFA data from an activated male gametocyte Fig. 6D column 4. First intensity data is shown with same color matching as Fig 6E. Then segmentation mask is overlayed on the mitochondrial marker channel and rotated showing the apparent presence of a single mitochondrion.

Supplementary movie 11

Description: Animation of IFA data from an activated male gametocyte Fig. 6E column 1. First intensity data is shown with same color matching as Fig 6E. Then segmentation mask is overlayed on the mitochondrial marker channel and rotated showing the clear presence of multiple or fragmented mitochondria.

Supplementary movie 12

Description: Animation of IFA data from an activated male gametocyte Fig. 6E column 2. First intensity data is shown with same color matching as Fig 6E. Then segmentation mask is overlayed on the mitochondrial marker channel and rotated showing the clear presence of multiple or fragmented mitochondria.
